# Supplementary material for: A New One-Tube Reaction Assay for the Universal Determination of Sweet Cherry (Prunus avium L.) Self-(In)Compatible MGST- and S-Alleles Using Capillary Fragment Analysis
Source: Int J Mol Sci. 2023 Apr 8;24(8):6931. doi: 10.3390/ijms24086931 (PMC10139232; doi:10.3390/ijms24086931)
Supplement: Supplementary file 1 [file ijms-24-06931-s001.zip › S-alleles_SupplMaterial_230329.pdf]

Supplementary material

Figure S1. The *P. avium* S54 allele from the 'Techlovicka' cultivar sequence analysis. A) genomic DNA alignment [OQ555802 for *P. avium* S54; JQ627790 for *P. virginiana* S2]; B) protein alignment [OQ555802 translation for *P. avium* S54; AFJ20682 for *P. virginiana* S2].

A)

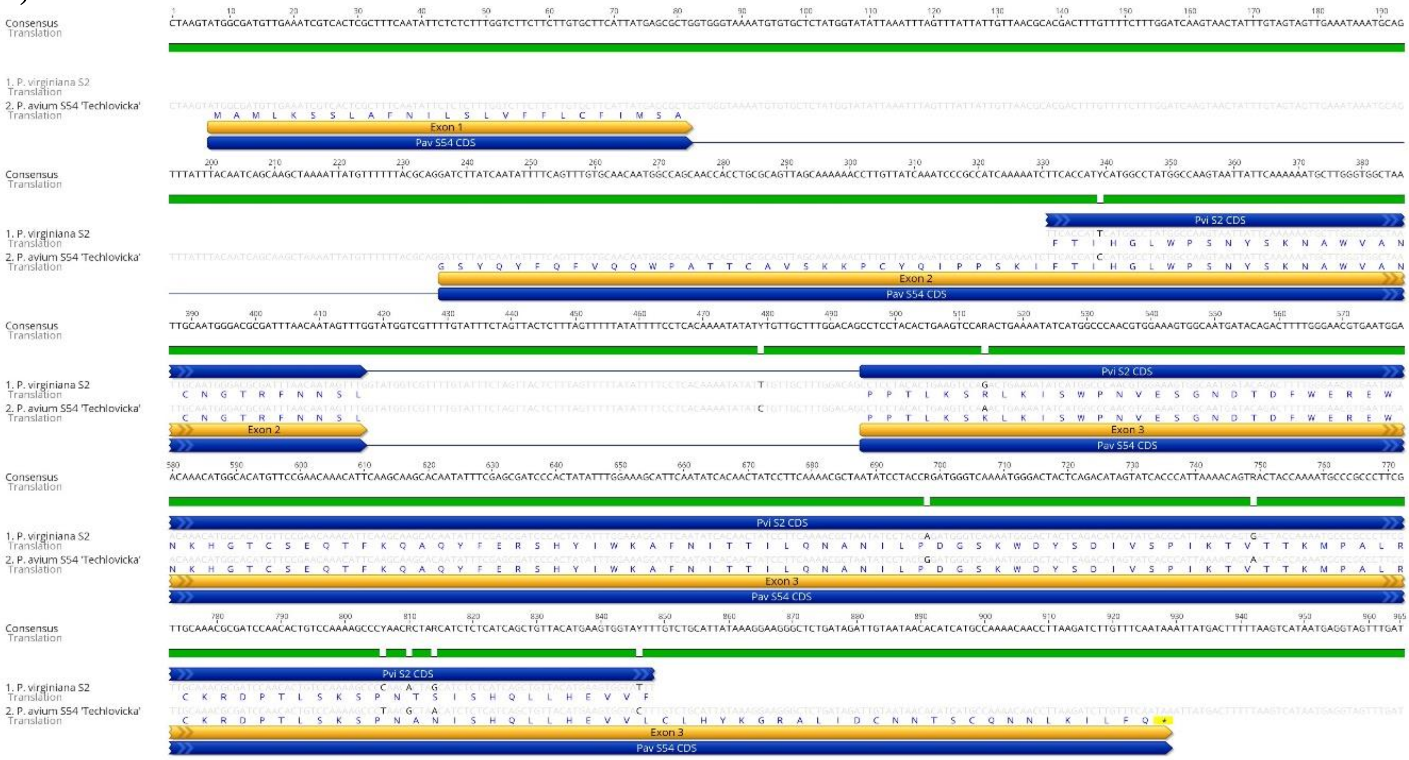

B)

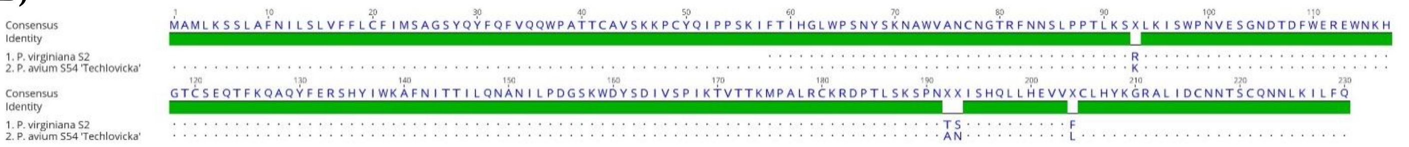

Figure S2. The *P. avium* S54 allele from the 'Techlovicka' cultivar phylogenetic tree. The S54 protein sequence was compared with BLAST records and the phylogenetic tree was constructed using Geneious Prime software. Branches are denoted by GenBank numbers.

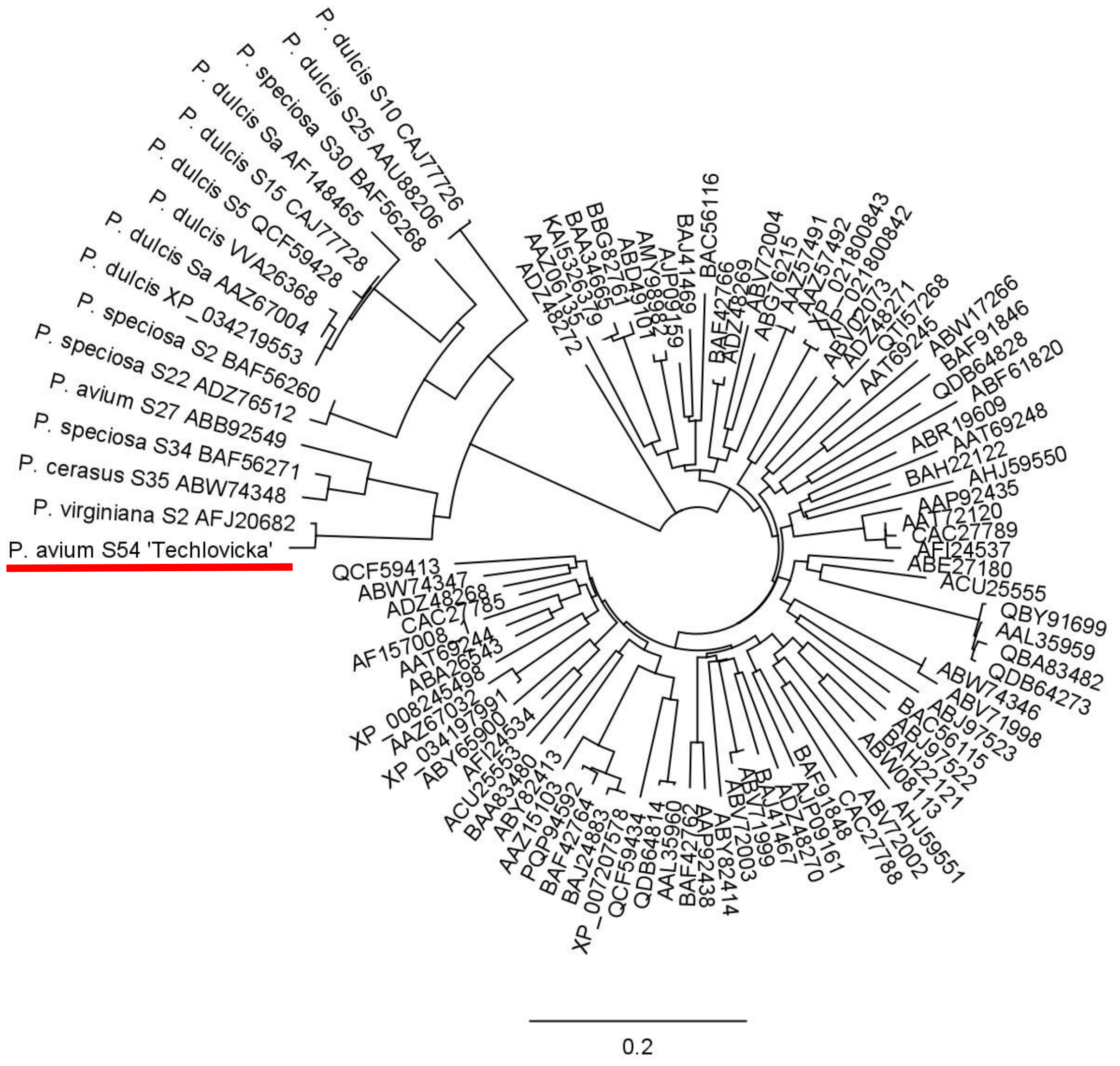

Figure S3. The S-RNase-Ex3-R primer binding site sequence analysis in selected S-alleles. The mismatched nucleotide is shown in bold red, and the region of the primer in the pink frame.

```
                                                                  S-RNase-Ex3-R
                                                               CGTGGAARGTGGCAATGATAC
S-RNase S1, Early Rivers, AJ635281             AAGAGATCTTGGCCCGACGTGGAAAGTGGCAATGATACAAGATTTTGGGAAGGCGAATGG   597
S-RNase S2, Early Rivers, AJ635283             AAGAGATCTTGGCCCGACGTGGAAAGTGGCAATGATACAAGATTTTGGGAAGGCGAATGG  1933
S-RNase S6, Colney, AJ635291                   CAGACATCTTGGCCGGACGTGGAAAGTGGCAATGATACAAAGTTTTGGGAAAGCGAATGG   306
S-RNase S7, Charger, AJ635268                  AAGATATCTTGGCCAGACGTGGAAAGTGGCAATGATACAAAATTTTGGGAAGGCGAATGG  1985
S-RNase S9, Inge, AJ635270                     AAGAGATCTTGGCCGGACGTGGAAAGTGGCAATGATACAAGGTTTTGGGAAGGCGAATGG   527
S-RNase S12, Schneiders Spaete Knorp., AJ635274 AAGAAATCTTGGCCGGACGTGGAAAGTGGCAATGATACAAAATTTTGGGAAGGCGAATGG  1496
S-RNase S13, Noble, AJ635276                   AAAAGATCTTGGCCCGACGTGGAAGGTGGCAATGATACAAAATTTTGGGAAGGCGAATGG   597
S-RNase S22, Kiechelsberger Kracher, DQ336138  AAGAAATCTTGGCCCGACGTGGAAAGTGGCAATGATACAAAATTTTGGGAAGGCGAATGG   332
S-RNase S24, Pico Colorado, CDS AY259112       CAGACATCTTGGCCGGACGTGGAAAGTGGCAATGATACAAAGTTTTGGGAAAGCGAATGG    92
```

Figure S4. Sequence analysis of MGST alleles. MGSTins: ´Cristobalina´; MGSTdel: ´Kronio´; MGSTwt: ´Satonishiki´

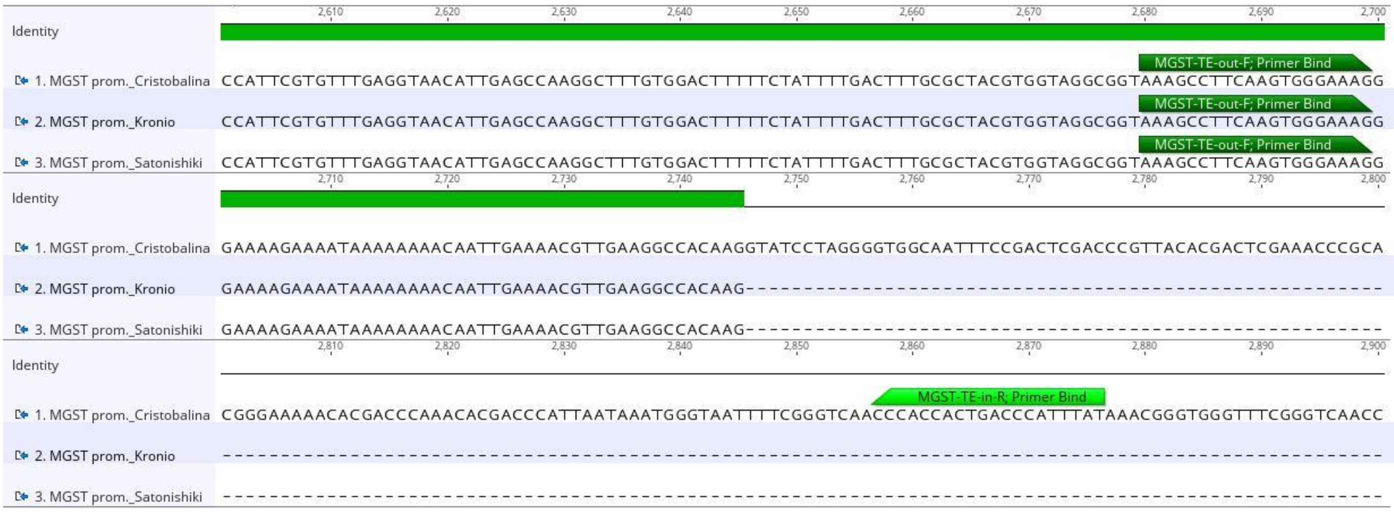

...

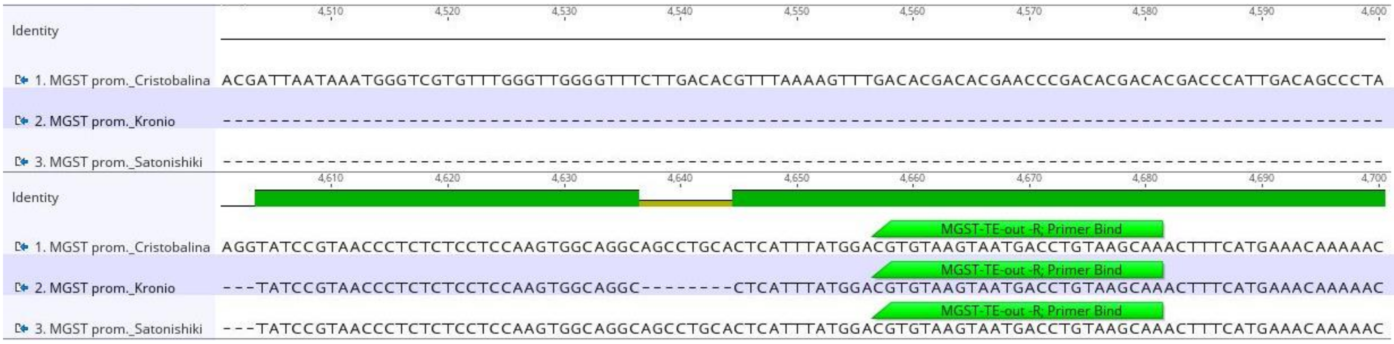

Figure S5. Outputs of fragment analyses of all tested S-alleles and MGST alleles. A) ´Charger´ (S1S7 MGSTwt); B) ´Feldes Fruehe Schwarze´ (S1S14 MGSTwt); C) HL18188 (S2S3´ MGSTwt); D) ´Buketova´ (S2S16 MGSTwt); E) Rtynska ptacka (S2S25 MGSTwt); F) ´Cristobalina´ (S3S6 MGSTwt/MGSTins); G) ´Winklerova´ (S3S22 MGSTwt); H) HL 13272 x ´Halka´ (S4S4´ MGSTwt); I) ´Viola´ (S4S12 MGSTwt); J) ´Techlovicka´ (S4S54 MGSTwt); K) ´Felicita´(S4´S9 MGSTwt); L) 1:1 mixed sample of ´Kronio´ (S5´S6 MGSTwt/MGSTdel) + ´Uriase de Bystrita´ (S5S12 MGSTwt); M) ´Uriase de Bystrita´ (S5S12 MGSTwt); N) ´Rita´ (S5S22 MGSTwt); O) ´Kronio´ (S5´S6 MGSTwt/MGSTdel); P) 437 NDR (S7S9 MGSTwt); Q) Italia 2 (S13S14 MGSTwt); R) P-HL-A (S13S25 MGSTwt).

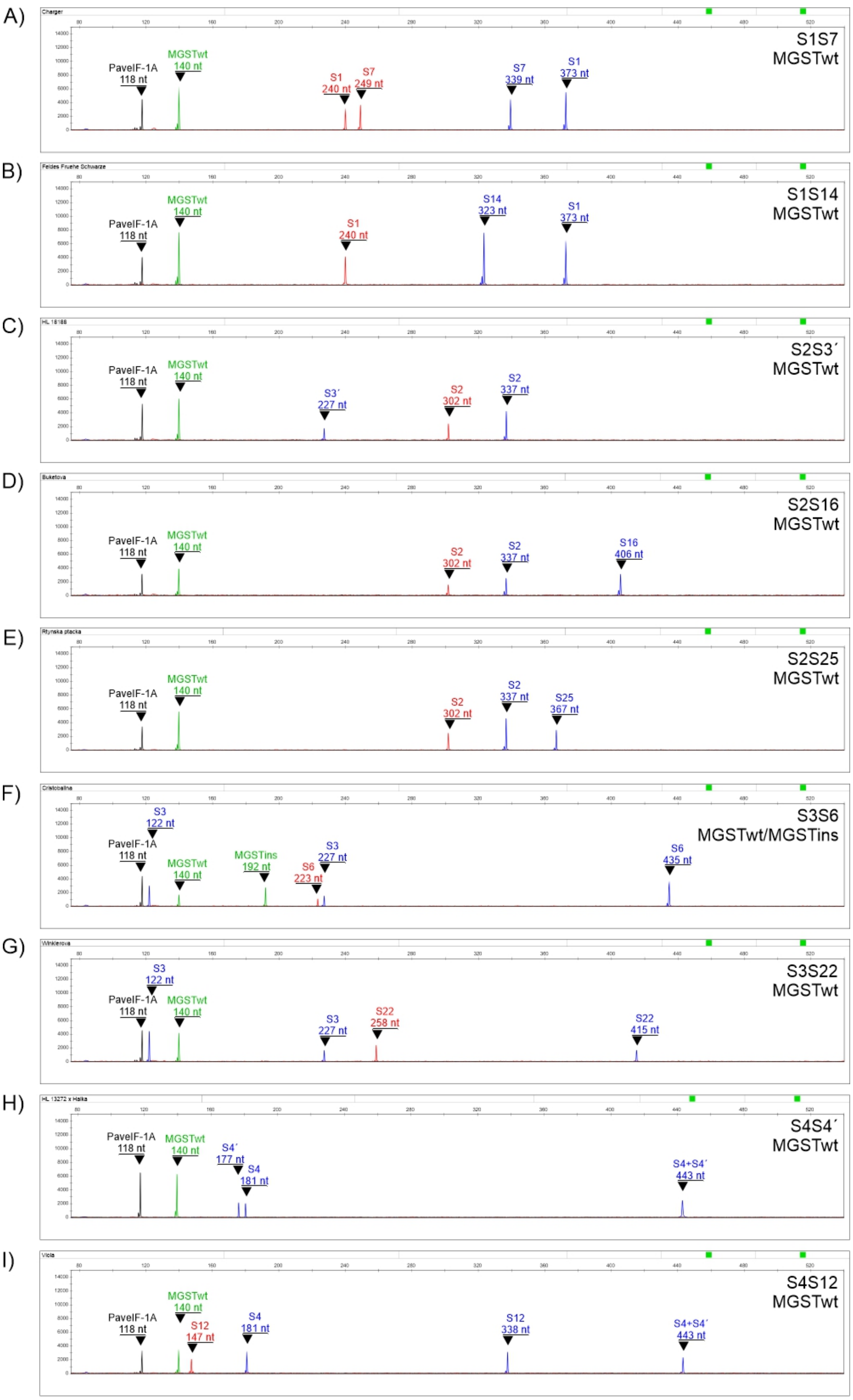

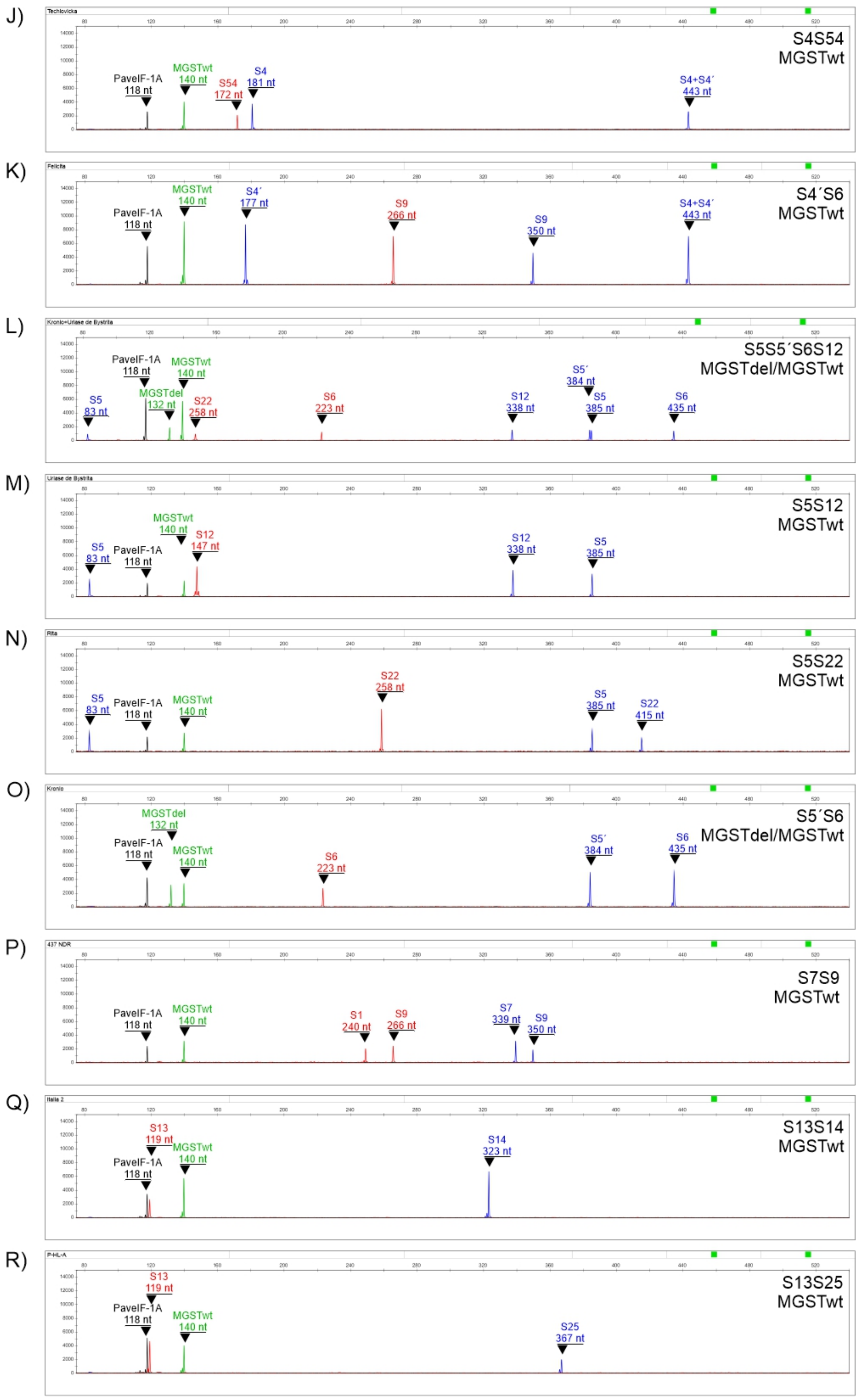
J)
PavelF-1A 118 nt
MGSTwt 140 nt
S54 172 nt
S4 181 nt
S4+S4´ 443 nt
S4S54 MGSTwt
K)
PavelF-1A 118 nt
MGSTwt 140 nt
S4´ 177 nt
S9 266 nt
S9 350 nt
S4+S4´ 443 nt
S4´S6 MGSTwt
L)
S5 83 nt
PavelF-1A 118 nt
MGSTdel 132 nt
MGSTwt 140 nt
S22 258 nt
S6 223 nt
S12 338 nt
S5´ 384 nt
S5 385 nt
S6 435 nt
S5S5´S6S12 MGSTdel/MGSTwt
M)
S5 83 nt
PavelF-1A 118 nt
MGSTwt 140 nt
S12 147 nt
S12 338 nt
S5 385 nt
S5S12 MGSTwt
N)
S5 83 nt
PavelF-1A 118 nt
MGSTwt 140 nt
S22 258 nt
S5 385 nt
S22 415 nt
S5S22 MGSTwt
O)
MGSTdel 132 nt
PavelF-1A 118 nt
MGSTwt 140 nt
S6 223 nt
S5´ 384 nt
S6 435 nt
S5´S6 MGSTdel/MGSTwt
P)
PavelF-1A 118 nt
MGSTwt 140 nt
S1 240 nt
S9 266 nt
S7 339 nt
S9 350 nt
S7S9 MGSTwt
Q)
S13 119 nt
PavelF-1A 118 nt
MGSTwt 140 nt
S14 323 nt
S13S14 MGSTwt
R)
S13 119 nt
PavelF-1A 118 nt
MGSTwt 140 nt
S25 367 nt
S13S25 MGSTwt

Figure S6: Outputs of fragment analyses in two cultivars using three different PCR reagents: Phusion Flash High-Fidelity PCR Master Mix (ThermoFisher Scientific), PCR Master Mix (2X) (ThermoFisher Scientific) and qPCR 2x Blue Master Mix (Top-Bio). A) ´Felicita´; B) ´Feldes Frühe Schwarze´.

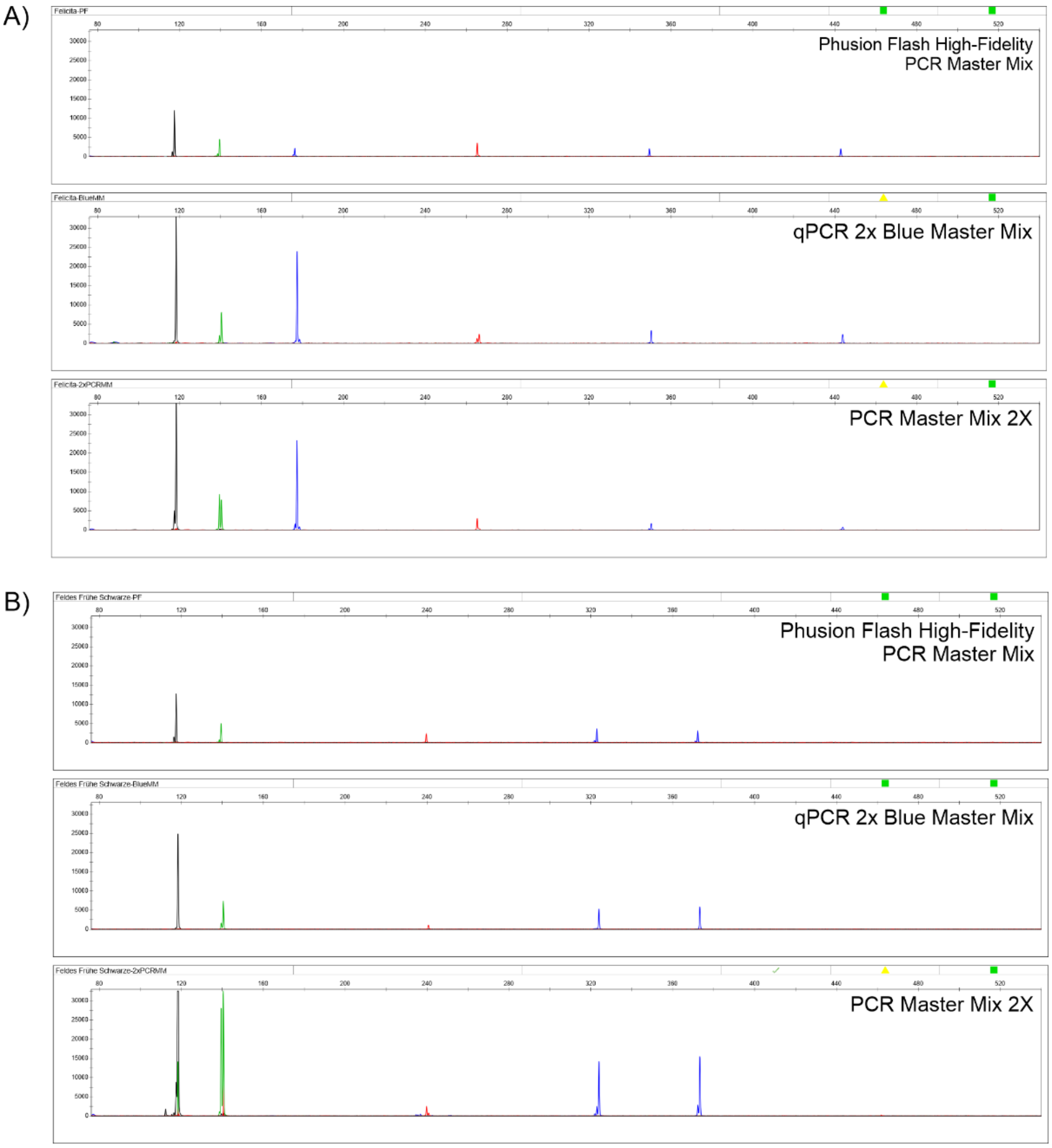

Table S1. The list of sweet cherry varieties used in the study.
Provided as a separate Excel file.

Table S2. Sequences used in the study. Reference sequences retrieved from the GenBank (National Center for Biotechnology Information (nih.gov)) or GDR (rosaceae.org).

	**S-RNase***	**SFB**	**MGST***	**PaveIF-1A**
**S1**	AJ635282; AJ635281	AB111518		
**S2**	AJ635284; AJ635283	AB111519		
**S3**	AY571663	AY571665		
**S3´**				
**S4**	AB028154.1	AB111521		
**S4´**		AY649873		
**S5**	AJ635290; AJ635289	AB111520		
**S5´**	EU077235	EU077237		
**S6**	AY571664; AJ635291	AY805051		
**S7**	EU035974; AJ635268	EU035976		
**S9**	AJ635271; AJ635270	DQ422809		
**S10**	JQ280519	AY805053		
**S12**	AY259115; AJ635274	AY805054.1		
**S13**	DQ385842; AJ635276	DQ385844		
**S14**	AJ635278; AJ635277			
**S16**	AJ635280; AJ635279	AY805056.1		
**S17**	JQ280528			
**S18**	JQ280533			
**S19**	DQ336139			
**S20**	AJ862659			
**S21**	AJ863119			
**S22**	EF429142; DQ336138			
**S23**	AY259114			
**S24**	AY259112			
**S25**	AY259113			
**S27**	DQ266439			
**S28**	DQ266440			
**S29**	DQ266441			
**S30**	DQ266442			
**S31**	DQ266443			
**S32**	DQ266444			
**S34**	JQ280525			
**S37**	JQ280522			
**S38**	JQ280516			
**S54**	OQ555802; this study			
**MGSTwt**			LG_3: 18395575 – 15400614**	
**MGSTdel**				
**MGSTins**			LC371380	
**PaveIF-1A**				LG_3: 15263431 – 15262737**

* In case sequences or their parts necessary for alignments were missing, sequences were generated in this study. See the text for more details.
** Retrieved from Sweet cherry Genome v1.0.
